# Comparison of Deep Learning Models for Cervical Vertebral Maturation Stage Classification on Lateral Cephalometric Radiographs

**DOI:** 10.3390/jcm10163591

**Published:** 2021-08-15

**Authors:** Hyejun Seo, JaeJoon Hwang, Taesung Jeong, Jonghyun Shin

**Affiliations:** 1 Department of Pediatric Dentistry, School of Dentistry, Pusan National University, Yangsan 50612, Korea; herrjoon@pusan.ac.kr (H.S.); tsjeong@pusan.ac.kr (T.J.); 2 Department of Oral and Maxillofacial Radiology, School of Dentistry, Pusan National University, Yangsan 50612, Korea; softdent@pusan.ac.kr; 3 Dental and Life Science Institute & Dental Research Institute, School of dentistry, Pusan National University, Yangsan 50612, Korea

**Keywords:** cervical vertebral maturation, classification, orthodontics, artificial intelligence, deep learning, convolutional neural networks, lateral cephalometric radiograph

## Abstract

The purpose of this study is to evaluate and compare the performance of six state-of-the-art convolutional neural network (CNN)-based deep learning models for cervical vertebral maturation (CVM) on lateral cephalometric radiographs, and implement visualization of CVM classification for each model using gradient-weighted class activation map (Grad-CAM) technology. A total of 600 lateral cephalometric radiographs obtained from patients aged 6–19 years between 2013 and 2020 in Pusan National University Dental Hospital were used in this study. ResNet-18, MobileNet-v2, ResNet-50, ResNet-101, Inception-v3, and Inception-ResNet-v2 were tested to determine the optimal pre-trained network architecture. Multi-class classification metrics, accuracy, recall, precision, F1-score, and area under the curve (AUC) values from the receiver operating characteristic (ROC) curve were used to evaluate the performance of the models. All deep learning models demonstrated more than 90% accuracy, with Inception-ResNet-v2 performing the best, relatively. In addition, visualizing each deep learning model using Grad-CAM led to a primary focus on the cervical vertebrae and surrounding structures. The use of these deep learning models in clinical practice will facilitate dental practitioners in making accurate diagnoses and treatment plans.

## 1. Introduction

Evaluation of the growth and development of children and adolescents is important for diagnosis and treatment in the field of medicine and dentistry [1,2]. There are various factors which correspond to a child’s growth and development status, such as height, weight, sexual maturation characteristics, chronological age, skeletal maturity, and dental development and eruption. Among them, evaluation of skeletal maturity is considered the most reliable method of determining growth and development status [2,3,4]. It aids in ascertaining the optimal time for dentofacial treatment based on skeletal maturity, and is used as a reliable indicator in forensic science and pediatric endocrinology [5,6].

Currently, hand–wrist radiograph analysis is considered to be the gold standard to evaluate skeletal maturity [7]. The evaluation of bone age using hand–wrist radiographs has the advantage of being able to evaluate the ossification onset of the ulnar sesamoid through the different types of bones detected in the area; therefore, it is widely used in the medical field [8,9].

Meanwhile, in the field of dentistry, many studies have been conducted to evaluate the growth stage using the cervical vertebral maturation (CVM) method in lateral cephalometric radiographs, which are primarily used for diagnosis in orthodontics as a predictable indicator of the growth stage [10,11,12]. This can reduce the radiation exposure from taking hand–wrist radiograph in growing children and adolescents [13].

However, as skeletal maturation is a continuous process, it might be difficult to differentiate the six stages of CVM for borderline subjects, and certain lateral cephalograms with a high level of radiographic ‘noise’ make staging difficult by affecting the clarity of the image [11,14]. Therefore, some studies believe that the CVM method lacks reliability and reproducibility due to the low agreement between observers [15]. Therefore, using the CVM method may be difficult for clinicians lacking technical knowledge and experience.

Recent progress in convolutional neural network (CNN) architectures using deep learning has led to the ability of direct inference, recognition, judgment, and classification [16]. They have been widely applied to medical image analysis. In particular, in the field of dentistry, CNNs perform tasks such as detecting, segmenting, and classifying anatomic structures (hard or soft tissue landmarks, teeth) and pathologies (dental caries, periodontal inflammation or bone loss, apical lesions etc.) [17]. Since the CNN technology imaging diagnosis time exceeds human ability and does not fatigue from repetitive tasks, its application in the medical field is highly likely to expand [18].

Currently, a fully automated system to predict skeletal age using deep learning on hand–wrist radiographs is widely used clinically, with high accuracy and visualization [19,20]. In contrast, CVM analysis studies on lateral cephalometric radiographs using deep learning differ in classification accuracy by about 80–90% due to differences in preprocessing techniques and deep learning models [21,22,23,24]. If CVM analysis is performed automatically on the lateral cephalometric radiograph, it can provide information on the skeletal maturity of growing children without specific training to clinicians and additional radiation exposure.

Class activation map (CAM) and gradient-weighted class activation map (Grad-CAM) technologies are being introduced to visualize deep learning models, which solve the shortcomings of the black box of deep learning models and provide ‘visual explanations’ to enhance their transparency [25,26]. However, in relation to CVM research, there have only been a few papers comparing and visualizing the performance of various CNN-based deep learning models so far.

Therefore, the purpose of this study is to evaluate and compare the performance of six state-of-the-art CNN-based deep learning models for CVM on lateral cephalometric radiographs, and implement visualization of CVM classification for each model using Grad-CAM technology.

## 2. Materials and Methods

### 2.1. Ethics Statement

This study was approved by the Institutional Review Board (IRB) of the Pusan National University Dental Hospital (Approval number: PNUDH-2020-026). The board waived the need for individual informed consent as this study had a non-interventional retrospective design and all the data were analyzed anonymously; therefore, no written/verbal informed consent was obtained from the participants.

### 2.2. Subjects

All patients aged 6–19 years, who underwent lateral cephalometric radiography (PM 2002 CCC, Planmeca, Helsinki, Finland) (78 kVp, 11 mA, and 1.5 sec) between 2013 and 2020 at the Pusan National University Dental Hospital, were included in this study. A total of 100 images were randomly extracted for each CVM stage from a pool of images in which the CVM stage had been read using Baccetti’s method by a radiologist with more than 10 years of experience. Thus, 600 images were collected. Chronological age was collected and calculated based on the date of filming and date of birth. All collected lateral cephalometric radiographs (1792 × 2392 pixels image, JPEG format) with a good visualization of the cervical vertebrae, including C2, C3, and C4 were included (Table 1).

### 2.3. Methods

**Figure 1:**
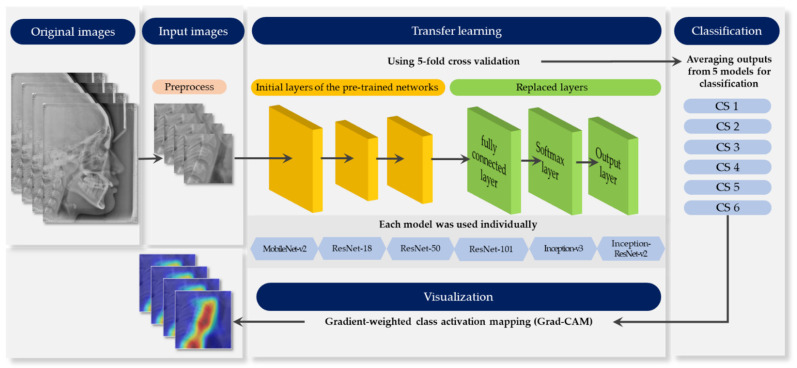
Schematic diagram representing the whole process.

Figure 1 provides an outline of the whole process. Each stage elaborates in the following sections.

#### 2.3.1. Pre-Process

Image patches of 550 × 550 pixels showing the inferior border of C2 to C4 vertebrae were manually cropped using the average anatomical position of the vertebrae in the lateral cephalometric radiographs. No further image processing, such as filtering or enhancing, was applied to the images to retain the original view of all information-containing soft tissues [23].

#### 2.3.2. Pre-Trained Networks

Six state-of-the-art convolutional neural networks, ResNet18, MobileNet-v2, ResNet-50, ResNet-101, Inception-v3, and Inception-ResNet-v2, were used for classifying CVM stages. The basic properties of the pre-trained networks are presented in Table 2.

To retrain the pre-trained networks for classification, the three layers were replaced with new layers adapted to the task. We replaced the final fully-connected layer, the softmax layer, and the classification layer with a new fully-connected layer of size 6 (the number of responses), new softmax layer, and new class layer.

#### 2.3.3. Data Augmentation

Various data augmentation techniques were used to reduce overfitting on deep learning models due to the small size of the dataset. The techniques for the training data set were performed through rotation from −7 to 7, scaling horizontally and vertically from 0.9 to 1.1, and translation horizontally and vertically from −5 to 5 pixels.

#### 2.3.4. Training Configuration

An NVIDIA Titan RTX graphic processing unit with cuDNN version 5.1 acceleration was used for network training. The models were trained for maximum 50 epochs, eight mini-batch sizes with the Adam optimizer [27], with an initial learning rate of e^−4^. A 5-fold cross validation was performed to evaluate performance. In this process, the entire data was evenly divided into five subsets; one set was a test set for validation, and the remaining four were used as training sets. After five iterations, the average output of five folds was obtained. All procedures were performed using MATLAB 2020a (MathWorks, Natick, MA, USA).

#### 2.3.5. Performance Evaluation

Multi-class classification metrics, accuracy (1), recall (2), precision (3), F1-score (4), and area under the curve (AUC) values from the ROC curve were used to evaluate the performance of the models.

(1) $$\mathrm{Accuracy}=\frac{\mathrm{TP}\;+\mathrm{TN}}{\mathrm{TP}\;+\mathrm{TN}+\mathrm{FP}+\mathrm{FN}}$$

(2) $$\mathrm{Recall}=\frac{\mathrm{TP}}{\mathrm{TP}\;+\mathrm{FN}}$$

(3) $$\mathrm{Precision}=\frac{\mathrm{TP}}{\mathrm{TP}\;+\mathrm{FP}}$$

(4) $$F1\text{-}\mathrm{score}=2\times \frac{\mathrm{Recall}\;\times \mathrm{Precision}}{\mathrm{Recall}+\mathrm{Precision}}$$

TP: true positive; FP: false positive; FN: false negative; TN: true negative.

#### 2.3.6. Model Visualization

Grad-CAM was visualized by weighing it on the activation map to determine the most relevant part in the classification result. Grad-CAM is based on the gradients of activation maps generated from the last convolutional layer for all CNN architectures [26].

## 3. Results

### 3.1. Classification Performance

**Figure 2:**
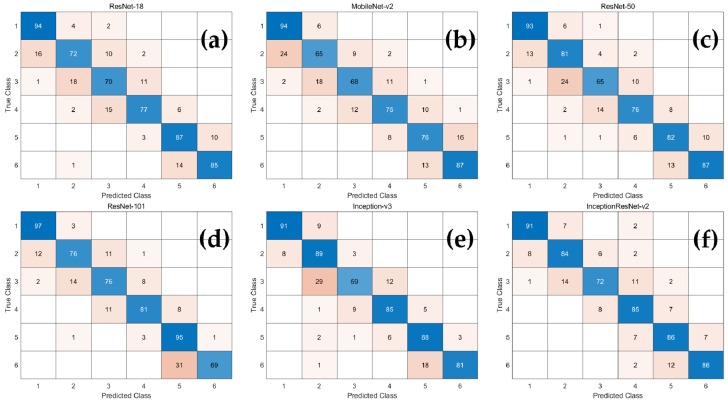
The confusion matrix of the CNN-based deep learning models for determination of CVM stages. (**a**) ResNet18; (**b**) MobileNet-v2; (**c**) ResNet-50; (**d**) ResNet-101; (**e**) Inception-v3; (**f**) Inception-ResNet-v2.

Accuracy, recall, precision, and F1-score were calculated using six multi-class confusion matrices (Figure 2) for each network. As demonstrated in Table 3, the average classification accuracy of all CNN-based deep learning models was over 90%. Among them, Inception-ResNet-v2 had relatively high accuracy, recall, precision, and F1-score, and those of MobileNet-v2 were low.

**Figure 3:**
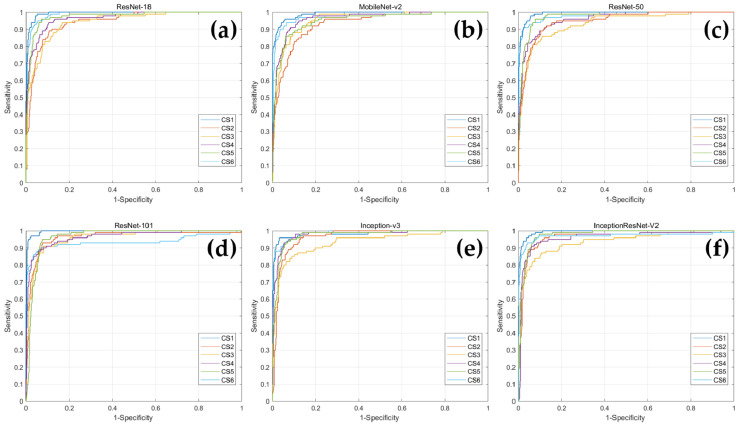
The ROC curves of the CNN-based deep learning models for determination of CVM stages. (**a**) ResNet18; (**b**) MobileNet-v2; (**c**) ResNet-50; (**d**) ResNet-101; (**e**) Inception-v3; (**f**) Inception-ResNet-v2.

In addition, ROC curves were drawn for each CVM stage corresponding to each deep learning model, and AUC values were obtained (Figure 3 and Table 4). When comparing the AUC values for each CVM stage within the network, Inception-v3 had the highest AUC value for CS 6, and the remaining five networks demonstrated highest value of AUC for CS 1. In MobileNet-v2, CS 2 had the lowest AUC value, ResNet-101 had the lowest AUC value in CS 6, and in the remaining four networks, CS 3 had the lowest AUC value.

### 3.2. Visualization of Model Classification

**Figure 4:**
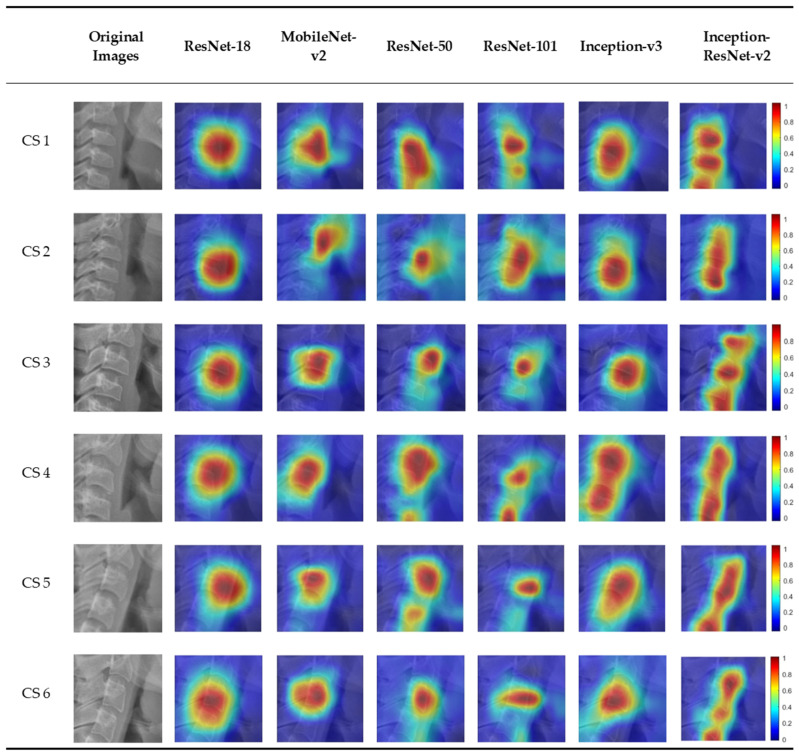
An example of Grad-CAMs of the CNN-based deep learning models.

Figure 4 shows the six CVM stages classified by the deep learning models overlapping the image of the heat map using Grad-CAM. In the activation map, blue (low) to red (high) indicates the degree of influence of decision from various sites. There was a slight difference in focus in classifying the six CVM stages for each model. Most of the deep learning models focus on or around the third cervical vertebra. Among them, Inception-ResNet-v2, which has the highest accuracy, classified CVM stages by focusing on several cervical vertebrae.

## 4. Discussion

The CVM method has inherent limitations because its guidelines are not strict and depend on the subjective evaluation of the observer [28]. In addition, lateral cephalometric radiographs may cause difficulties in evaluation due to image distortion depending on the angle and posture of the patient [29]. Therefore, the clinician should be specially trained to be able to make a satisfactory evaluation using the CVM method [30]. Therefore, the deep learning algorithm using AI will help clinicians to make an accurate assessment and reduce variables [31]. It also helps to reduce manual errors and the time required for diagnosis in computer-assisted analysis of dental radiographs, which leads to high efficiency and accuracy [32]. Therefore, automatic analysis of CVM assessment using deep learning will help clinicians to easily assess the stages of growth.

In this study, the cervical vertebrae shown in lateral cephalometric radiographs could be classified into six stages with over 90% accuracy using all CNN-based deep learning models, and it was visualized using Grad-CAM. Among them, Inception-ResNet-v2 scored the highest with 94.06%, and MobileNet-v2 scored the lowest with 91.22%. The number of parameters in a CNN network can increase the amount of learning. Among the six CNN networks, Inception-ResNet-v2, with the number of parameters as 55.9 × 10^6^, showed the highest accuracy, and MobileNet-v2, with the smallest number of parameters as 3.5 × 10^6^, showed the lowest accuracy. The rest of the networks also showed a positive correlation between the number of parameters and accuracy. In addition, although it is generally known that the deeper the network depth, the higher is the accuracy [33], this study did not reveal that depth and accuracy are proportional in networks with different structures. In ResNet architecture, the higher the network depth, the higher was the accuracy. In other network architectures, ResNet-18 with shallower depth showed better performance than Mobilenet-v2 with deeper depth. This can be attributed to features such as multiple skip connections in ResNet-18 which prevent loss of information between layers. Hence, it could be regarded as achievable sufficient learning despite the fewer number of layer [34]. Additionally, based on the fact that Inception-ResNet-v2 recorded the highest performance, it is necessary to learn a large number of features to learn the CVM stage from lateral cephalometric radiographs. It was also verified that a network with a deep and complex structure is required for learning.

Regarding accuracy based on the stage in the network using AUC, the AUC value was lowest in CS 3 as compared to other stages. Some studies reveal that CS 3 was the lowest in intra-rater absolute agreement (50% or less) compared to other CS [35]. A previous study on CVM classification using deep learning showed that CS 3 and 4 recorded relatively lower accuracy (72%) than other stages [24], although the accuracy differs in this study. The CS 3 stage, being a pubertal stage, contains a growth peak [5]. Therefore, it is speculated that variations in the cervical vertebrae increase due to an active growth pattern, which leads to low accuracy. 

Classification of the CNN model by the CAM technology permits visualization and greater transparency of the model by identification of discriminative regions [25]. Reconstruction of the global average pooling (GAP) layer is required for CAM, which leads to the disadvantage of CNN-based architecture not being free. Grad-CAM technology, which does not require GAP structure or reconstruction of the CNN model permits a wider range of application generalization of the model by equating the bias of the dataset [26]. Grad-CAM will help provide the basis for human judgment to trust AI through visualization of deep learning models. This study confirmed the areas important for classification among the six deep learning models in the CVM stage classification process, using Grad-CAM, and identified the characteristic activation map for each deep learning model (Figure 4). There was a difference in focus according to the heat map for each model. The highest classification accuracy of Inception-ResNet-v2 is attributed to the fact that it focuses on several cervical vertebrae. Most deep learning models classify CVM stages by focusing on a specific area of the cervical vertebrae, which showcases the difference in classification by different clinicians.

Although the training time varied for each deep learning model, all models computed CVM classification within 0.1 s for a single image (Table 5). In addition, further studies including performance comparison between humans and deep learning model might help establish an efficient and optimal deep learning model for clinical application. If a deep learning model is used as an auxiliary means for maturation stage classification of cervical vertebrae after taking lateral cephalometric radiographs, it would help shorten the diagnosis time of clinicians with little experience with maturation classification.

A limitation of this study was the small number of 600 lateral cephalometric radiographs that were used for training a deep learning model with data augmentation. In future, the use of more high-quality data and development of better-performing CNN architectures may aid the creation of models with more than 95% performance. Another limitation was that the difficulty in evaluation of the cervical vertebrae on the lateral cephalometric radiographs due to surrounding structures. The use of a deep learning-based approach to medical image segmentation has recently received greater attention and improved the accuracy of diagnosis [36]. The possibility of an automatic diagnosis on lateral cephalometric radiographs with segmentation of the cervical vertebrae will provide clinicians with accurate information on skeletal maturity.

## 5. Conclusions

This study classified the CVM stages on lateral cephalometric radiographs using six state-of-the-art CNN-based deep learning models. All deep learning models showed more than 90% accuracy, and among them, Inception-ResNet-v2 performed relatively best. In addition, as a result of visualizing each deep learning model using Grad-CAM, the cervical vertebrae and surrounding structures were mainly focused. The use of deep learning models in clinical practice will aid dental practitioners in making accurate diagnoses and treatment plans.

## Figures and Tables

**Table 1:** Descriptive statistics of the subjects’ age by cervical stage.

CVM Stage	Numbers	Mean Age (Years) ± SD
CS 1	100	7.27 ± 1.17
CS 2	100	9.41 ± 1.60
CS 3	100	10.99 ± 1.28
CS 4	100	12.54 ± 1.08
CS 5	100	14.72 ± 1.58
CS 6	100	17.65 ± 1.69
Total	600	12.10 ± 3.52

CVM: cervical vertebral maturation; SD: standard deviation; CS: cervical stage.

**Table 2:** Properties of pre-trained convolutional neural networks (CNNs).

Network Model	Depth	Size (MB)	Parameter (Millions)	Input Image Size
ResNet-18	18	44.0	11.7	224 × 224 × 3
MobileNet-v2	53	13.0	3.5	224 × 224 × 3
ResNet-50	50	96.0	25.6	224 × 224 × 3
ResNet-101	101	167.0	44.6	224 × 224 × 3
Inception-v3	48	89.0	23.9	299 × 299 × 3
Inception-ResNet-v2	164	209.0	55.9	299 × 299 × 3

**Table 3:** Accuracy, precision, recall, and F1-score corresponding to the CNN-based deep learning models. Values presented in the table are in the format of mean ± standard deviation.

	Accuracy	Precision	Recall	F1-Score
ResNet-18	0.927 ± 0.025	0.808 ± 0.094	0.808 ± 0.065	0.807 ± 0.074
MobileNet-v2	0.912 ± 0.022	0.775 ± 0.111	0.773 ± 0.040	0.772 ± 0.070
ResNet-50	0.927 ± 0.025	0.807 ± 0.096	0.808 ± 0.068	0.806 ± 0.075
ResNet-101	0.934 ± 0.020	0.823 ± 0.113	0.837 ± 0.096	0.822 ± 0.054
Inception-v3	0.933 ± 0.027	0.822 ± 0.119	0.833 ± 0.100	0.821 ± 0.082
Inception-ResNet-v2	0.941 ± 0.018	0.840 ± 0.064	0.843 ± 0.061	0.840 ± 0.051

**Table 4:** CVM stage classification performance by the AUC corresponding to the CNN-based deep learning models.

	CS 1	CS 2	CS 3	CS 4	CS 5	CS 6
ResNet-18	0.993	0.945	0.944	0.967	0.976	0.989
MobileNet-v2	0.990	0.934	0.954	0.964	0.953	0.980
ResNet-50	0.992	0.949	0.934	0.959	0.975	0.983
ResNet-101	0.996	0.962	0.959	0.965	0.965	0.935
Inception-v3	0.983	0.964	0.935	0.978	0.974	0.987
Inception-ResNet-v2	0.994	0.961	0.935	0.959	0.975	0.969

**Table 5:** Processing time details corresponding to different deep learning models.

	ResNet-18	MobileNet-v2	ResNet-50	ResNet-101	Inception-v3	Inception-ResNet-v2
Training time	9 min, 30 s	21 min, 10 s	22 min, 20 s	47 min, 25 s	41 min, 30 s	119 min, 40 s
Single image testing time	0.02 s	0.02 s	0.02 s	0.03 s	0.03 s	0.07 s

## Data Availability

Restrictions apply to the availability of these data. Data used in this study was obtained from Pusan National University Dental Hospital and are available with the permission of the Institutional Review Board of Pusan National University Dental Hospital, Pusan National University, Dental Research Institute.
